# CSC Radioresistance: A Therapeutic Challenge to Improve Radiotherapy Effectiveness in Cancer

**DOI:** 10.3390/cells9071651

**Published:** 2020-07-09

**Authors:** María Auxiliadora Olivares-Urbano, Carmen Griñán-Lisón, Juan Antonio Marchal, María Isabel Núñez

**Affiliations:** 1 Department of Radiology and Physical Medicine, University of Granada, 18016 Granada, Spain; auxiou@correo.ugr.es; 2 Biopathology and Regenerative Medicine Institute (IBIMER), Centre for Biomedical Research, University of Granada, 18100 Granada, Spain; glcarmex@gmail.com; 3 Department of Human Anatomy and Embryology, Faculty of Medicine, University of Granada, 18016 Granada, Spain; 4 Instituto de Investigación Biosanitaria de Granada (ibs.GRANADA), 18012 Granada, Spain; 5 Excellence Research Unit “Modeling Nature” (MNat), University of Granada, 18016 Granada, Spain

**Keywords:** radiation resistance, CSC intratumoral radiosensitivity heterogeneity, accelerated repopulation, CSC niche, CSC metabolism, signaling pathways, tumor microenvironment

## Abstract

Radiotherapy (RT) is a modality of oncologic treatment that can be used to treat approximately 50% of all cancer patients either alone or in combination with other treatment modalities such as surgery, chemotherapy, immunotherapy, and therapeutic targeting. Despite the technological advances in RT, which allow a more precise delivery of radiation while progressively minimizing the impact on normal tissues, issues like radioresistance and tumor recurrence remain important challenges. Tumor heterogeneity is responsible for the variation in the radiation response of the different tumor subpopulations. A main factor related to radioresistance is the presence of cancer stem cells (CSC) inside tumors, which are responsible for metastases, relapses, RT failure, and a poor prognosis in cancer patients. The plasticity of CSCs, a process highly dependent on the epithelial–mesenchymal transition (EMT) and associated to cell dedifferentiation, complicates the identification and eradication of CSCs and it might be involved in disease relapse and progression after irradiation. The tumor microenvironment and the interactions of CSCs with their niches also play an important role in the response to RT. This review provides a deep insight into the characteristics and radioresistance mechanisms of CSCs and into the role of CSCs and tumor microenvironment in both the primary tumor and metastasis in response to radiation, and the radiobiological principles related to the CSC response to RT. Finally, we summarize the major advances and clinical trials on the development of CSC-based therapies combined with RT to overcome radioresistance. A better understanding of the potential therapeutic targets for CSC radiosensitization will provide safer and more efficient combination strategies, which in turn will improve the live expectancy and curability of cancer patients.

## 1. Introduction

For many types of cancer, cancer stem cells (CSCs) represent a subpopulation with specific surface markers and functional properties including self-renewal capacity, long-term repopulation potential, and tumor initiation and progression capacity, which make these cells different from the bulk tumor cells [1,2].

Tumor heterogeneity is responsible for the differing sensitivities of tumor cells to cancer treatment including radiotherapy (RT), which explains why tumor subpopulations are not equally affected by this treatment. This is likely to be true for most tumors and scientific evidence in different cancers has shown that CSCs are resistant to conventional treatments including ionizing radiation (IR) [3,4,5,6,7,8,9]. Other factors to be considered are the coupling between tumor heterogeneity and heterogeneity of the tumor microenvironment as well the changes the tumor undergoes over time, during cancer progression and RT.

About 50% of cancer patients receive RT, normally conventional RT with photons, during the course of their treatment [10]. Theoretically, tumors can be controlled if a sufficiently high dose of radiation is delivered to eradicate the CSC population in a tumor. Nevertheless, in the clinical practice, the deleterious effects on surrounding normal tissue limit the radiation dose [11]. Moreover, despite technological advances in RT and the new treatment strategies implemented, resistance to RT and recurrence of the disease still represent major limitations in radiation oncology. Resistance of CSCs to radiation can be either intrinsic (or primary) or acquired, the latter leads to the development of adaptive responses induced by the irradiation itself [12,13]. Resistance to conventional RT is involved in RT failure, metastasis, cancer relapses, and a poor prognosis in cancer patients [14]. A plethora of studies has been done to specifically target the DNA damage response (DDR) to obtain selective cancer cell radiosensitization [15,16]. Nevertheless, despite the efforts made to overcome radioresistance, the mechanisms behind its development are still not fully understood.

The intrinsic radioresistance of CSC is present within the cell even before the treatment has started and it can be attributed to several factors [17]. In addition to these mechanisms, several studies have illustrated that the activation of survival signaling pathways, such as anti-apoptotic Bcl-2 and PI3K/Akt/mTOR, also contribute to the radioresistance of CSCs) [18,19].

Radioresistance of CSC reveals the need for reevaluation of the underlying mechanisms of the response of solid tumors to conventional and new RT with a specific focus on CSCs. Some experimental works have suggested different molecular mechanisms associated to CSC resistance to conventional therapy [1,17]. Radioresistance of breast CSC (BCSC) has been associated with a lack of oxidative stress due to the increased ability of CSCs to remove free radicals and to active DNA repair mechanisms [3]. Other authors have shown repopulation of BCSC after RT through the activation of WNT/β-catenin signaling, which promotes self-renewal [4]. Radioresistance of CSC from mucoepidermoid carcinoma has also been associated to the activation of the NFκB signaling pathway [18].

In order to overcome CSC resistance to conventional therapies, different strategies such as high-dose and high-linear energy transfer (LET) RT, immunotherapy, gene therapy, molecular inhibition, and combination therapy have been widely investigated. The identification of molecular targets that control CSC can drive the development of new drugs able to eradicate and prevent the growth of new CSCs in patients. This will help prevent metastasis and tumor relapse with a reduction of morbidity and toxicity, and ultimately improving the outcomes in cancer patients [19].

Although many patients are still treated with conventional RT, other advanced RT techniques have been developed such as stereotactic body radiation therapy (SBRT) that uses with accuracy high doses of radiation delivered to target local tumors. SBRT has been applied to the treatment of several cancers [20,21,22] with promising results for early lung cancers, with local control rates above 80% [20,23]. In addition to SBRT, particle-beam therapy has demonstrated excellent therapeutic outcomes in various types of cancer with a growing number of patients undergoing this therapy [24]. Nevertheless, no sensible theory can explain as of yet the association between the physical characteristics of high LET radiation or high radiation dose and their biological effects. This information would be essential to understand CSC radioresistance and to improve patient care.

Since the present work deals with CSC radioresistance, we review the main characteristics of this subpopulation, its plasticity and tumor heterogeneity associated with the number of CSCs within the tumor. In order to better comprehend CSC radioresistance, an understanding of the molecular mechanisms associated to the development of this radioresistance is essential. In addition, it is critical to consider the microenvironment response to RT as this response varies depending on the CSC niche. The ways to increase tumor radiocurability and the 5Rs of RT are also discussed as well as the different CSC-targeting therapies. Lastly, future perspectives aimed at the use of RT and combined therapies are also described. Considering the involvement of CSCs in radioresistance, tumor relapse, and metastasis, there is a demanding need to find new therapeutic strategies to eradicate this aggressive cell subpopulation. Thereby, this review offers insight into the mechanisms involved in CSC radioresistance and into the development of CSC-specific therapies combined with RT, some of which are being currently implemented in the clinical setting. This will allow a better understanding of the safety and efficacy of these combination therapies.

## 2. CSC Subpopulation

### 2.1. Biological Characteristic of CSCs

A major challenge in the fight against cancer is the ability of tumor cells to metastasize and cause relapse. This can be explained by the tumor heterogeneity and the presence of cells with stemness properties within the tumor bulk termed cancer stem cell (CSCs), tumor initiating cells (TICs), or tumor stem cells (TSCs). Solid tumors mimic aberrantly developed organs and tissues and are composed of heterogeneous cell populations including neoplastic cells, supporting vascular cells, inflammatory cells, and fibroblasts. Only the small subpopulation of CSCs is long-lived and has extensive self-renewal and tumorigenic capacities [25,26,27].

Several hypotheses have been proposed to explain the tumorigenic process. One of them is the hierarchical CSC model, which implies the existence of a small subpopulation of cells inside the tumors at the top of the hierarchy with self-renewal and asymmetric cell division properties. This subpopulation is therefore able to proliferate and maintain the growth indefinitely, unlike the other cells within the tumor [26,28,29,30]. Although the origin of CSCs is not clear, they seem to appear in most types of cancer including breast, pancreas, or brain, among others, and what seems clear is their involvement in metastasis, tumor recurrence, and drug resistance [25,29,30,31,32].

More recently, the CSC dynamic hypothesis postulates that the CSC phenotype is flexible and can be influenced by the tumor microenvironment (TME). Thus, after asymmetric division of CSCs they differentiate and give rise to the heterogeneous cell populations within the tumor. Under the influence of TME, which includes neighboring stromal cells, vascular and lymphatic networks, and soluble factors, differentiated tumor cells can dedifferentiate into CSCs. This theory implies the plasticity of tumor cells at several stages of maturation and the close relationship between tumor cells and their surrounding non-tumor cells [33].

A better understanding of the CSC characteristics that contribute to tumor progression and metastasis will improve the rational use of established therapeutic regimes and the search of novel strategies targeting CSCs and TME.

### 2.2. CSC Plasticity

**Figure 1:**
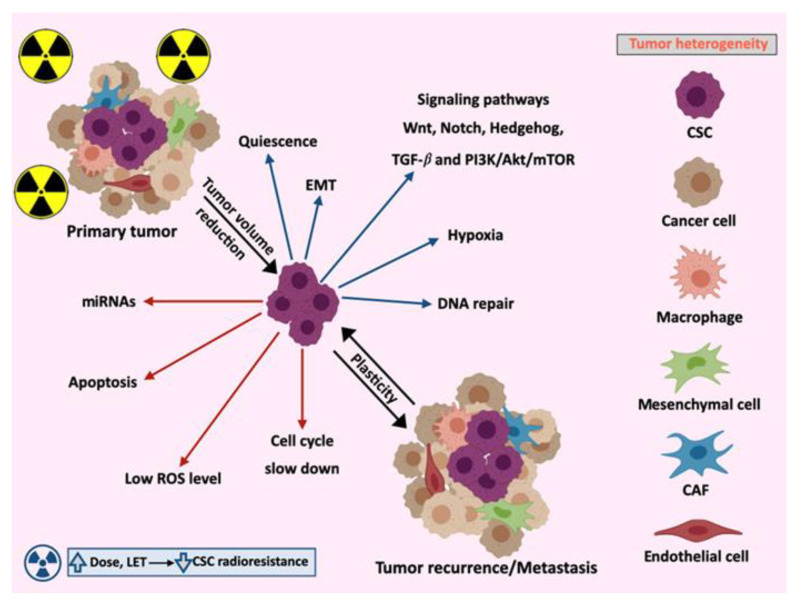
Targeting cancer stem cells to overcome radioresistance. Tumor heterogeneity leads to differing responses to anticancer therapies of the cell subpopulations within a tumor. Conventional radiotherapy eliminates more differentiated cancer cells, but specific cancer stem cell (CSC) phenotypes can evade the cytotoxic effects of this treatment and start an accelerated repopulation of the tumor therefore promoting cancer recurrence and metastasis. CSC radioresistance can be attributed to several factors: (1) activation of DNA repair mechanisms; (2) capacity to maintain a quiescence state; (3) increased activation of DNA damage checkpoints; (4) repopulation and reoxygenation of hypoxic areas in the tumor; (5) increased ability to remove free radicals; (6) high plasticity associated to the epithelial mesenchymal transition process; (7) activation of survival signaling pathways (Wnt, Notch, Hedgehog, anti-apoptotic Bcl-2, TGF-β, and PI3K/Akt/mTOR); and (8) upregulation of different biological processes. In order to eradicate CSC more efficiently higher absorbed doses and higher linear energy transfer (LET) radiation using different radiation qualities other than a photon would be necessary to control the radioresistant CSCs.

The first strong in vivo evidence of CSCs came from studies in human leukemia by Bonnet and Dick in 1997. Isolated leukemic stem cells from human acute myeloid leukemia were transplanted into non-obese diabetic mice with severe combined immunodeficiency (NOD/SCID) to investigate their capacity to initiate leukemias. These leukemia-initiating cells were defined by the expression of the cell surface antigen CD34 and they exhibited self-renewal, differentiation, and proliferation capacities similar to normal hematopoietic stem cells [34,35]. Self-renewal, which is responsible for tumorigenesis, and differentiation ability in specific lineages of stem cells are regulated by environmental signals present in the niche of these cells. Normal stem cells and CSCs share common signaling pathways that regulate the self-renewal activity, including Wnt, Notch, and Sonic Hedgehog. In addition, other signaling molecules such as PTEN also play important roles in the regulation of CSC growth and other important signaling pathways like the transforming growth factor-ß (TGF-ß). Some of these routes are frequently deregulated in cancer and can play a crucial role in in cancer stem-like cells. Wnt/β-catenin and Notch pathways enhance the self-renewal activity during leukemia stem cell progression and are also involved in the regulation of normal and malignant breast stem cell populations (Figure 1) [27,29,30,36].

Another property of CSCs is the capacity to enter a quiescence state in the G0 phase for long periods where they do not divide, and which allows them to survive anticancer treatments (Figure 1). Quiescent cells may reenter the cell cycle even decades after the initial treatment, which would explain tumor recurrences in colon or breast cancer [26,37].

Several studies have provided evidence of the plasticity of both CSCs and non-CSCs and their ability to undergo phenotypic transitions in response to the exposure to specific microenvironmental factors. Dedifferentiation is a process by which non-CSC tumor cells retain their CSC phenotype and therefore their plasticity, which is highly dependent on the epithelial–mesenchymal transition (EMT) process. As described above the TME plays a critical role in the plasticity affecting the CSC state, from the origin of the CSCs to their metastatic potential. CSC plasticity is also related to tumor type and cell context [35,38]. CSCs differentiate into the differentiated cell populations within the tumor, which may in turn dedifferentiate under the regulation of stromal cell-derived factors in the TME. The cells in the surrounding tissue and the non-CSCs located on the edges of the tumor mass will be the most directly exposed to the factors from the TME. In fact, a few non-CSCs have the capacity to initiate a primary tumor that promotes the malignant transformation of the cells in the surrounding tissue to form the TME. The action of the secretome, which includes extracellular vesicles, cytokines, miRNAs, or mRNAs released by the cells in the surrounding tissue, promotes the conversion of the phenotype of the differentiated tumor cells in the tumor edges into undifferentiated CSCs. These CSCs can invade adjacent areas and enter the vascular and lymphatic systems facilitating distant spread [39].

As mentioned above, CSCs are closely related to the EMT process, which is essential during embryonic development for the conversion of epithelial cells into mesenchymal cells and are likely to be the basis for plasticity, tumor progression, and metastasis. The EMT process involves the loss of epithelial markers such as claudin and E-cadherin and the acquisition of mesenchymal markers such as vimentin and N-cadherin. Moreover, overexpression of EMT transcription factors promotes not only a mesenchymal-migratory phenotype, but also the tumor-initiating potential of cell lines [26,30,40]. In fact, EMT markers are found in almost all tumor types and this is associated with poor prognosis [41].

The TME plays a key role in the initiation of the EMT process by the secretion of many pro-inflammatory cytokines and factors including TGFβ1, interleukine-6 (IL6), interleukine-8 (IL8), epidermal growth factor (EGF), fibroblast growth factor (FGF), hepatocyte growth factor (HGF), platelet-derived growth factor (PDGF), and vascular endothelial growth factor (VEGF) [42]. In a previous work we showed that the secretome from mesenchymal stem cells (MSCs), considered tumor stromal cells, can induce the enrichment of tumor subpopulations with CSC-like phenotype, behavior and properties by promoting a specific cytogenetic profile, mainly a chromosome 17 alteration (17q25). In fact, IL-6 and HGF increased the stemness of CSC subpopulations from primary and established tumor cell lines and induced in vivo tumorigenic capacity [43]. In addition, these stromal cell-derived factors participate in the reprogramming of differentiated tumor cells toward a CSC state therefore increasing the tumorigenic capacity, aggressiveness, and metastasis by promoting EMT [44,45,46].

In a previous work on breast cancer we have demonstrated that in obese fat the interaction between stromal cells and cancer cells increases the secretion of proinflammatory cytokines and generates tumor-driving loops that promote CSC invasion and metastasis [47]. This increased a cytokine secretion by immature adipocytes in obese fat surrounding breast tumor increases the migratory capacity of CSC therefore promoting metastasis [47,48].

### 2.3. Tumor Heterogeneity

There is a great variability in the molecular characteristics and behavior between tumors affecting the same organ, a fact that is referred as “intertumor heterogeneity”. This results in a large number of tumor subtypes that are classified according to their molecular profile, morphology, and expression of specific markers, which also implies differences in the therapeutic response. Moreover, the results obtained from the examination of one single tumor site in metastatic disease might be not valid for a different tumor site in the same patient [49]. This intertumor heterogeneity between patients could result in an intrinsic radiation response as measured by a molecular signature [50]. In addition, the coexistence of different subpopulations of cancer cells within a tumor contribute to intratumor heterogeneity, which can also lead to treatment resistance due to the wide variations in the response of the different cell subpopulations [51]. Several theories have been proposed to explain inter- and intratumor heterogeneity as well as their clinical relevance [52].

Recent studies have demonstrated that tumor heterogeneity depends on the number of CSCs, which contributes to tumor growth. Intratumor heterogeneity involves different levels of cytogenetic markers, gene and protein expression, genetic mutations, and epigenetic regulation. CSCs require complex cell-to-cell and matrix interactions in order to create the heterogeneity needed by the TME to maintain tumor growth [35,53,54]. The relation and connection between CSCs and other tumor cells are essential to tumor progression, for example, MSCs inside tumors may act on carcinogenesis by favoring the generation of CSCs, invasion, and metastasis [55]. CSCs use their stemness properties to survive stress, chemotherapy, and RT, and experimental data have shown the great heterogeneity in tumor radiosensitivity [35,50].

In addition, there is also CSC heterogeneity, which is very complex. Numerous techniques have been developed to identify the specific markers of CSC heterogeneity, but this has proved difficult. Primarily, the most common biomarkers to identify CSCs are: (i) the determination of the side population (SP) by the flow cytometry sorting method that explores the ability of CSC to efflux the Hoechst 33342 via the ATP-binding cassette (ABC) transporters, which are multidrug resistance proteins; (ii) the overexpression of surface markers such as CD133 or CD44; (iii) the high activity of the enzyme aldehyde dehydrogenase (ALDH); (iv) the colony-forming ability; and finally (v) the in vivo tumorigenic ability [30,56,57].

## 3. Molecular Mechanisms Involved in CSCs Radioresistance

The intrinsic radioresistance of CSCs present even before the treatment has started can be attributed to several factors [58]. In addition to these mechanisms, several studies have demonstrated that the activation of signaling pathways related to survival such as Bcl-2 and PI3K/Akt/mTOR also contribute to CSC radioresistance (Figure 1) [59,60].

### 3.1. Signaling Pathways

The activation of essential signaling pathways for embryonic development and adult tissue homeostasis are closely related in the resistance to cancer treatment and the faster repopulation of CSC after or during RT. In fact, repopulation of tumors is a main factor responsible for the failure of conventional fractionated courses of RT. The Wnt/β-catenin pathway, Notch signaling, Hedgehog pathway, TGF-β, and PI3K/AKT/mTOR pathway are involved in these processes.

#### 3.1.1. Wnt/β-catenin

The Wnt signaling pathway is important for self-renewal, dedifferentiation, apoptosis inhibition, and metastasis and it has also been found to be involved in CSC radioresistance by increasing the levels of activated β-catenin after radiation [26,30]. This pathway promotes the proliferation of CSCs and their stability in niches after RT, causing radioresistance. Furthermore, this survival pathway is related to DNA repair and cell cycle checkpoints playing an important role in the regulation of CSC apoptosis, which further promotes CSC survival and radioresistance [61,62]. Wnt signaling also plays an important role in the dedifferentiation of CSCs by SOX2 expression. An EMT process is activated by Wnt with an increase in β-catenin levels in the nucleus, acting as a promoter of tumor metastasis and FOXO3a knockdown enhancing β-catenin accumulation [63,64]. Thus, the radiosensitivity of cancer cells can be enhanced by inhibiting the Wnt/β-catenin pathway.

#### 3.1.2. Notch

The activation of Notch signaling is related with more aggressive tumors. This is a well-known CSC pathway that is highly conserved in a wide range of human tumors and it is involved in the upregulation of self-renewal and repressing/downregulation of differentiation. A high Notch pathway activity has been correlated with poor prognosis and radioresistance [30]. The activation of the Notch pathway by radiation might be part of the acute response to this treatment, thereby initiating the transcription of gene products that promote progression into the S-phase of the cell cycle [61,65]. Irradiation induces the expression of Notch receptor ligands on the surface of non- tumorigenic cells and activation of Notch signaling, which makes quiescent CSCs enter into the cell cycle and acquire EMT and self-renewal properties. Moreover, Notch activity is activated via γ secretase (GS) and it is targeted and inhibited using γ-secretase inhibitors (GSIs) and blocked by γ-secretase inhibitors (GSIs), which inhibit CSC and disease recurrence cancer by slowing down angiogenesis and promoting apoptosis of tumor cells [64,66,67].

#### 3.1.3. Hedgehog (Hh)

The Hedgehog (Hh) pathway is mainly involved in the development of organs in most animals. A histological correlation has been found between Hh signaling and tumor recurrence, which supports the role of Hh signaling in relapse of advanced tumors [30]. A potential role of the Hh signaling in irradiation-induced EMT has also been suggested as blockage of Hh activity that inhibits the expression of EMT stimulating genes. These events and components mediate radioresistance and irradiation-induced tumor repopulation in vivo [65,68]. Cancer cells produce Hh ligands responsible for the reprogramming of cancer-associated fibroblasts (CAFs) to produce a supportive niche for maintaining CSC stemness. Nutritional deprivation (stress) of tumor cells results in Hh ligand activation, which also contributes to dedifferentiation of these cells into CSCs [64,66].

#### 3.1.4. TGF-β

The TGF-β signaling pathway is involved in many cellular processes in organisms and in the developing embryo, such as cell proliferation and differentiation, apoptosis, and homeostasis. TGF-β is produced by the mass of the non-tumorigenic, radiosensitive cancer cells and activated by radiation [30,61]. In CSCs, TGF-β also plays an important role in cell proliferation and self-renewal. The role of TGF-β in the EMT process in cancer is well understood, the TGF-β-induces EMT and the resulting CSC phenotype and even it is influenced by specific microenvironmental signals, which leads to conversion of non-CSCs into tumor cells with stemness properties [64,66]. TGF-β, a major promoter of CSC stemness, has been shown to play critical roles in radioresistance by activating DNA repair pathways. In contrast, the inhibition of TGF-β signaling has been shown to increase radiosensitivity and radiation-induced tumor growth delay [65,68].

#### 3.1.5. PI3K/AKT/mTOR

The PI3K/Akt/mTOR pathway plays an important role in cell growth and proliferation and is often dysregulated in human tumors. AKT promotes survival, metastasis, and radioresistance by modulating downstream effectors, including caspases, nuclear factor-κB (NF-κB), and the mammalian target of rapamycin (mTOR) family [61,69]. The PI3K/AKT/mTOR pathway promotes antioxidative processes and quiescence in CSCs and plays an important role in radiation-induced autophagy. Recently, it has been demonstrated that dual targeting of PI3K and mTOR overcome radioresistance and improve treatment efficacy in various cancer cell types [64,69,70].

### 3.2. Apoptosis

The natural mechanism for programmed cell death is the apoptosis, which is a highly regulated process that plays a critical role in development and homeostasis by eliminating unnecessary cells. Several conditions can induce the apoptotic pathway, such as DNA-damaging agents, anticancer drugs, ROS, UV irradiation, TNF-α, and bacterial toxins [71]. Apoptosis signaling begins in one of two ways: the intrinsic apoptotic signaling pathway mainly integrates various signals in cancers, like the Bcl-2-like family of proteins initiates a pathway through the mitochondria and cytochrome c, after which caspase 9 is activated and the extrinsic apoptotic signaling such as the Fas receptor and TNF-related apoptosis inducing ligand (TRAIL) initiates a caspase activation [72,73,74]. CSCs use different mechanisms of apoptosis evasion. One of the main mechanisms is autophagy and glucose uptake, which promotes the growth and survival of CSCs in hypoxic and low-glucose TMEs [66]. Immune surveillance and dormancy lead to equilibrium between cancer cell apoptosis and proliferation. Immunotherapy uses tumor antigens to change this equilibrium and eliminate tumor cells [75]. After irradiation, surviving CSCs can repopulate the tumor because they have greater capacity of activating the DNA damage checkpoints than the non–CSCs. Activation of the Akt pathway, Oct-4 and the cell division cycle protein 20 (CDC20) confer to CSCs apoptosis resistance to RT via the apoptosis inhibitor gene, survivin [66].

### 3.3. Cell Cycle

Cell irradiation results in a delayed progression between the stages of the cell cycle. This occurs through the activation of DNA damage checkpoints, which are specific points in the cell cycle where the cell can be blocked or slowed. The DNA damage response (DDR) activates four distinct checkpoints (chks) at different points within the cell cycle in response to irradiation. These checkpoints are G_1_/S, S, early G_2_, and late G_2_. The ataxia-telangiectasia mutated protein is the apical kinase thought to regulate, through phosphorylation of hundreds of substrates, the global cell responses to double-strand breaks (DSBs), including the coordination of DSB repair events and the activation of cell cycle checkpoints and DNA repair to prevent genomic instability [76,77,78,79,80]. In a large number of tumor cells, one or more of these chks are disabled due to genetic changes and other alterations that occur during tumorigenesis. Alteration in genes that mediate checkpoint activation will result in the failure to delay progression in the cell cycle in response to irradiation. The presence or absence of chks will affect the redistribution of cells in the cell cycle after irradiation. Cells in mitosis/G_2_ are more sensitive to radiation and those in the late S phase, more radioresistant. Since not only radioinduced damage varies during the cell cycle but also the DNA repair capacity changes, this may indirectly affect cell sensitivity to subsequent doses of radiation. Thus, radiation exposure induces a redistribution of the cell in the cycle resulting in an accumulation of cells in the S phase [76,77]. Abnormal expression of cell cycle-related proteins may promote CSC proliferation after radiation. CSCs exhibit an attenuated activation of p53 after radiation-induced DNA damage, which results in impaired cell cycle arrest. Additionally, in CSCs, the family chks ½, which is activated after genotoxic stress, is a potential modulator to resistance to DNA targeting agents since it induces arrest of the cell cycle to allow DNA repair. These kinases have higher basal and inducible activities in CSCs than in non-stem cells. Finally, in breast cancer RT results in quiescent CSCs reentering into the cell cycle [61,65,81].

### 3.4. Epithelial–Mesenchymal Transition (EMT)

One of the “hallmarks of cancer” is the activation of invasion and metastasis mainly due to the epithelial–mesenchymal transition (EMT) program [26,40].

During EMT, epithelial cells acquire migratory properties, lose epithelial markers such as E-cadherin adherent proteins, and express mesenchymal markers such as vimentin and N-cadherin. Since Snail and Twist regulate E-cadherin and N-cadherin expression is regulated by Snail, activity is required for EMT initiation and by Twist, which intervenes in the maintenance of EMT. Thus, the Snail and Twist family promotes stemness properties and recurrent tumors have been found to express reduced E-cadherin and increased N-cadherin [61]. Other proteins, genes, and factors have been studied in relation to the acquisition of EMT properties like FOXO3a, whose silencing promotes an EMT-like phenotypic transition [63]. It has been demonstrated that overexpression of EMT transcription factors promotes the mesenchymal-migratory phenotype and also enhances the tumor-initiating potential of cell. EMT is also implicated in the incorporation of tumor cells into blood and lymph vessels, commonly named circulating tumor cells (CTC). The presence of CTC has been associated with an increased risk of tumor recurrence and distant metastasis [82].

Tumor cells undergoing radiation-induced EMT increase their motility and invasive capabilities in several cancers including breast, lung, liver, and glioma [68] The evidence suggests that ionizing radiation can increase the development of metastasis in the primary tumor and in normal tissues [83,84,85]. Even sublethal radiation doses have shown to increase the invasive and migratory capabilities glioma cells [86,87].

ROS have also been involved in radiation-induced EMT, via the activation of transcription factors (Snaіl, HIF-1, ZEB1, and STAT) activated by signaling pathways, including those of TGF-β, Wnt, Hedgehog, Notch, G-CSF, EGFR/PI3K/Akt, and MAPK. Activation of EMT leads cells to acquire stemness and to deregulate their metabolism, although this is still under debate. EMT, stemness, and oncogenic metabolism are known to be associated with radiotherapy resistance [68]. Irradiation can trigger EMT and enhance the migration, invasion, and radioresistance of hypopharyngeal carcinoma cells through the AKT/GSK-3β/Snail axis [88]. Some authors have suggested that targeting radiation-induced EMT might enhance RT efficacy by inhibiting the reactivation of dormant hypoxic CSCs and promoting antitumor immune responses. Thus, this strategy would be effective in regulating radioresistant CSC proliferation and maximizing the antitumor immunity of RT [89].

### 3.5. MicroRNAs

MiRNAs are a class of small non-coding RNAs that bind to target mRNAs and cause mRNA dysregulation and translation inhibition. MiRNAs regulate survival, self-renewal, apoptosis, and DNA damage in CSCs. The same miRNA can target different genes in different tumors and therefore assume different functions. Researchers have demonstrated that miRNAs can regulate tumor radioresistance. Moreover, the study of exosome-derived miRNAs opens a new horizon as they can serve as potential diagnostic and therapeutic biomarkers of RT [64,90,91]. In this respect, there seems to be a strong link between miRNAs, CSCs, and EMT. The miRNAs of the miR-200 family have been found to modulate EMT and promote the invasion of cancer cells. miRNAs also target the p21 tumor suppressor gene. Three abundant miRNA clusters, miR-200b-200a-429, miR-200c-141, and miR-183-96-182 are induced by p21, and the expression of miR-34, which inhibits EMT in epithelial cancer cells via targeting Snail, is induced by p53 [61,66]. Two well-known miRNAs, the miR-let-7 family and miR-21, are related to oncogenic and tumor suppressor functions by targeting multiple signaling pathways and by regulating a number of biological processes and are upregulated in CSCs conferring radioresistance [92,93]. The ataxia-telangiectasia mutated gen activates HIF1a through different mechanisms, which in turn transcriptionally regulates the expression of miR-210, miR-21, and miR-34a. miR-210 is highly radioresistant and overexpressed under hypoxic conditions [80,94]. Lastly, depending on the radiation dose delivered the expression of miRNAs varies in breast cancer and can be used as a biomarker in a personalized treatment [95].

## 4. CSCs and Microenvironment

CSCs are a small portion of cells within tumors and important research targets because of their tumorigenic potential and stemness properties [96,97]. CSCs are found mainly in hypoxic niches, with low pH and with less nutrients [75,98], where they can initiate, maintain, and disseminate tumors [64,96]. CSCs are also involved tumor recurrence and metastasis due to their radio- and chemoresistance ability [96,99]. This resistance ability is a consequence of their recovery capacity conferred by intrinsic properties of the cells themselves and extrinsic properties from the TME [64].

TME is made up of a network of stromal cells, immune cells, cancer-associated fibroblasts (CAFs), vascular endothelial cells, and a series of secreted molecules that assemble the extracellular matrix (ECM) [61,100]. Some immune cells and CAFs produce factors such as growth factors, cytokines, and chemokines that regulate the phenotype and function of tumor-resident cells. These factors also seem to promote primary tumor growth and metastasis by changing the TME for metastasis, CSC maintenance, and self-renewal [68]. Among others, these tumor and stroma-derived growth factors and cytokines include interleukines (IL), chemokines (CXC), tumor necrosis factor-alpha (TNF), epidermal growth factor (EGF), vascular endothelial growth factor, and fibroblast growth factor (FGF) [101,102]. In this respect, IL-6 protects CSCs against radiation-induced DNA damage and apoptotic death and IL-23 facilitates radioresistance by activating Wnt/Notch-mediated G0/1 phase arrest. Additionally, the inhibition of IL4 or IL10 results in the radiosensitivity of colorectal cancer cells [103,104]. The VEGF is involved in tumor growth and metastatic potential, associated with radiation by upregulation of HIF-1α and NF-κB [64,65]. On the other hand, the EGFR modulates DNA repair after radiation and therefore has been linked to a worse prognosis [105].

There is evidence that ECM receptors can be used to aggregate CSCs and induce drug resistance [30] and that TME can affect tumor development and metastasis [30,106]. For this reason, the interactions between tumor cells and non-malignant cells within the TME are fundamental in the pathophysiology of cancer [100]. Furthermore, TME is responsible for the various genetic and epigenetic characteristics of the different types of tumors [64].

TME is not only essential for the shape, function, development, and growth of tissues [96,100,107], but also for maintaining the optimal conditions of CSCs [39,61,96,108] and promoting tumor growth [109]. Within the TME, CSCs reside in specific areas known as CSC niches, where they can survive, self-renew, reactivate [30,61,75,110], and maintain their plasticity due to the autocrine and paracrine signals provided by the TME [111,112,113].

According to the available evidence on glioblastoma, cells in the tumor periphery have low levels of HIF1α, low proliferation rate, and greater angiogenesis. On the other hand, the cells of the tumor nucleus exhibit high levels of HIF1α and very low proliferation rates. Finally, the cells of the intermediate region show a high proliferation rate, higher levels of expression of VEGF, Glut1, and carbonic anhydrase IX (CAIX), and form neurospheres under hypoxic conditions [108].

During tumor growth, most solid tumors undergo hypoxia, which is responsible for a more aggressive tumor behavior [108,114] and resistance to radio- and chemotherapy because hypoxia promotes the CSC properties associated with EMT, maintains their plasticity, stimulates early EMT by suppressing E-cadherin and induces unfolded protein response (UPR) [61,64,111,114]. The effect of hypoxia on cells is controlled by a family of transcriptional regulators, hypoxia-inducible factors (HIF) [61,108,109], which can also inhibit cellular apoptosis [30]. HIFs are composed of an oxygen-dependent HIF-α subunit and an oxygen-independent HIF-β subunit [108,109].

Hypoxia can affect the TME and there is evidence that pharmacological induction of tumor hypoxia promotes CSC accumulation and tumor metastasis. Little is known about the molecular pathways involved, although CSC accumulation has shown that the tumor suppressor gene PTEN is downregulated. Hypoxia-driven PTEN deregulation also causes HIF-1α activation and mTOR signaling along with EMT induction [114].

The development of hypoxic niches is also due to reduced vascularity in the tumor [114]. However, the hypoxic condition itself stimulates pro-angiogenic factors for the formation of new blood vessels from the pre-existing vasculature [109]. Angiogenesis induction is a tumor characteristic and is considered one of the first steps in the development of invasive cancers [100,108]. In the TME, angiogenesis is induced by CSCs to allow greater absorption of the oxygen and nutrients needed to survive and proliferate, so CSCs are usually located close to the blood vessels in order to access the blood circulatory system [109,115].

Vascular homeostasis is controlled by pro- and anti-angiogenic factors, and angiogenesis is initiated when pro-angiogenic signaling predominates. Pro-angiogenic factors include VEGF, fibroblast growth factor 2 (FGF-2), platelet-derived growth factor (PDGF), angiopoietins, ephrins, apelin (APLN), and chemokines [109]. VEGF is one of the strongest inducers of angiogenesis and includes VEGF-A, VEGF-B, VEGF-C, VEGF-D, and placental growth factor (PlGF) [109,116]. During tumor angiogenesis, tumor cells produce VEGF to induce endothelial cell proliferation and survival. VEGF also mediates cell invasion, which is promoted by the expression of matrix metalloproteases (MMPs) [109].

MMPs are a family of zinc-dependent endopeptidases that are expressed by tumor cells or by surrounding stromal cells during tumor progression, and whose main function is remodeling the ECM and release growth factors that promote tumor progression, metastasis, and tumor-associated angiogenesis [96,109,117]. Furthermore, MMPs regulate cell–cell interactions and are involved in other physiological functions and in different carcinogenic processes such as tumor growth, angiogenesis, avoidance of apoptosis, response to inflammation, degradation of the basement membrane and invasion, modulation of the EMT process, formation of premetastatic niches, and metastasis [96,118]. MMPs are regulated by endogenous metalloproteinase inhibitors (TIMPs), and the MMPs/TIMPs ratio often determines the degree of degradation of ECM proteins and tissue remodeling [117]. Some authors have proposed MMP-targeted therapy to prevent second malignancies after breast radiotherapy [119].

### 4.1. Crosstalk between CSCs and Their Niches

As already described, the TME is a complex network of cells and soluble factors that regulates extracellular acidification, inflammation, activation of MMPs, and chemoresistance [106]. Within the TME, CAFs are one of the most abundant stromal cell types in several tumors [100]. The origin of CAFs is not entirely clear and four hypotheses have been proposed: (1) EMT; (2) transfer of fibroblasts in the stroma of the host; (3) transdifferentiation of perivascular cells; and (4) differentiation of MSCs derived from bone marrow. It is also unclear if CAFs adopt different functions depending on their origin [30], but they do participate significantly in tumorigenesis [30,100] through cell–cell interactions [30,120] and through the secretion of soluble factors that feed tumor cells [106]. Some of the pro-tumorigenic functions of CAFs are increased tumor cell invasion, improved EMT by Hedgehog signaling, ECM remodeling by MMPs, stimulation of tumor initiation in premalignant cells, increased profile of CSCs, promotion of migration and metastasis, and increased therapeutic resistance [30,100,121].

Additionally, tumor cells in the TME are able to control the immune response by increasing the amount of myeloid-derived suppressor cells, tumor-associated neutrophils (TANs), dendritic cells, and tumor-associated macrophages (TAMs) [111]. TAMs, like CAFs, participate in tumorigenesis by promoting genetic instability and tumor growth, nurturing CSCs, paving the way to metastasis and suppressing the protective adaptive immunity [122,123]. TAMs can perform different functions by differentiating into different subtypes through a process called polarization, which involves bidirectional interaction between the tumor microenvironment and macrophages. These TAM subtypes are classically activated macrophages (M1) and alternatively activated macrophages (M2) [122]. M1 macrophages have antitumor effects, produce pro-inflammatory factors, and participate in the immune defense against external pathogens. Instead, M2 are considered the true TAMs and promote angiogenesis, immunosuppression, and metastasis [30,109,122].

### 4.2. CSCs Niches in the Primary Tumor and Metastasis

As previously mentioned, the CSC niches [30,61,75,110] have specific conditions of hypoxia, acidity, and low glucose levels [75,98]. Under unfavorable environmental conditions, CSCs can enter a dormant or quiescent state and remain in the G0 phase. The hypoxic environment promotes survival and proliferation of tumor cells. An acidic pH is toxic to normal cells and promotes degradation of the ECM [110]. The low glucose level is due to the inhibition of glycolysis (a process required by tumor cells for energy production) by the acidic pH, which results in increased oxidation of fatty acids and oxidative phosphorylation. These metabolic reactions provide the low energy levels required by dormant CSCs [75,111]. Tumor relapse and metastasis require CSCs leaving the quiescent state and survive and proliferate by evading the innate immune response. Several studies have described that the TME provides the necessary signals to regulate both the input and the output of the CSCs to quiescence [110].

The metastasis process involves the remodeling of the components of the ECM, the migration of tumor cells from the primary site into the surrounding stromal tissue, the intravasation through the blood and lymph vessels, and extravasation out from the capillaries [75,110]. Metastatic niches, like primary niches, require cellular and molecular components that regulate the survival and proliferation of tumor cells. In this respect, MMPs are essential for ECM organization and tumor cells produce a series of soluble factors, cytokines, and chemokines that support the formation of the premetastatic niche through the recruitment of TAMs, TANs, myeloid-derived suppressor cells (MDSC), and regulatory T cells (Treg) [110]. The crosstalk between CSCs and their niche is bidirectional, which means that the niche can influence CSCs and these in turn can modify the niche in response to cancer therapies [61,75]. The close relationship between CSCs, EMT, and metastatic capacity is due to cell–cell interactions mediated by adhesion molecules such as CD44 and N-cadherin [75].

### 4.3. CSCs and Microenvironment in Response to Radiation

RT has been one of the mainstays of cancer treatments for more than 100 years [96,124]. It has been broadly demonstrated that crosstalk between cancer cells and tumor stromal cells may regulate CSC fate and resistance to RT.

**Figure 2:**
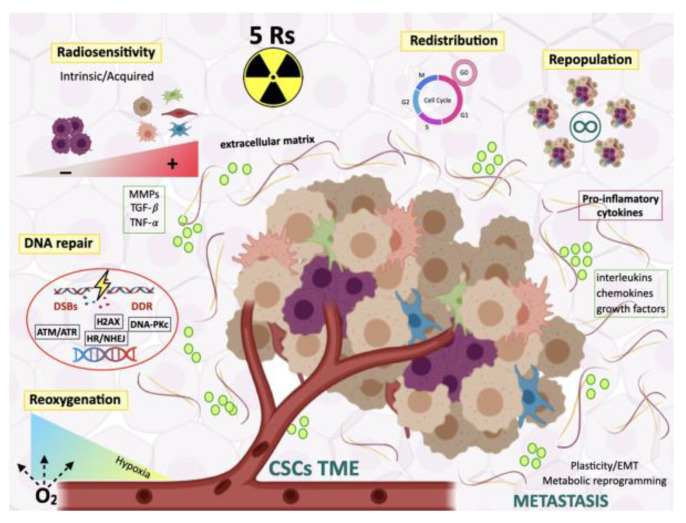
The five Rs of radiation biology and the tumor microenvironment influence on tumor response to radiotherapy. Radiation-induced DNA damage, such as double-strand breaks (DBSBs) trigger a DNA damage response. Three kinases (ATM, ATR, and DNA-PKc) phosphorylate the H2AX histone that plays an essential role in the response to ionizing radiation. Radiosensitivity: intrinsic and acquired radioresistance of cancer cells and particularly of CSCs is important to determine the outcome to radiotherapy. Repair mechanisms of sublethal damage after radiation exposure involve homologous repair (HR) and non-homologous end joining (NHEJ). Repair takes place after cell cycle blockage mediated by ATM, ATR, CHK1, and CHK2, among others. Redistribution of cells in the cell cycle affects their radioresistance. Cells in the late-S phase are more resistant and cells in the G2/M-phase are more sensitive to radiation. The time between two fractions allows resistant cells in the S-phase to redistribute into phases where cells are more radiosensitive. Repopulation: CSCs not eradicated by irradiation are involved in the accelerated repopulation of the tumor. Reoxygenation: cells in hypoxic niches within the tumor are more radioresistant. Reoxygenation between radiation fractions is important to increase tumor cell killing, which contributes to radiosensitization of hypoxic areas increasing the efficacy of the radiation treatment. Radiotherapy induces the production of pro-inflammatory cytokines and other molecules (growth factors, metalloproteases -MMPs-, TNFα, TGF-β, interleukins, etc.) in the tumor microenvironment that are involved in the interaction between CSCs and their niche and between CSCs and non-CSCs determining CSC radiosensitivity. CSC metabolic reprogramming associated to cell plasticity and epithelial to mesenchymal transition process is involved in metastasis. In CSCs having extensive cell plasticity, niche signals will reinstruct stem cell properties to progenitor or differentiated cells after CSC loss, which will result in tumor regeneration and therapy failure. Blocking niche signals that specifically sustain CSC identity will be more effective and improve the therapeutic efficacy by inhibiting plasticity and CSC regeneration.

Ionizing radiation can modify TME and its components through different mechanisms including CAF activation, growth factor release, changes in protein expression, states similar to EMT, induction of hypoxia, or inflammation associated with infiltration of immune cells (Figure 2) [61,125]. However, some of these mechanisms and the damage caused by irradiation can trigger CSC resistance to cancer treatments. Microenvironmental signals, metabolic adaptation, expression of transcription factors, and epigenetic alterations are involved in therapeutic resistance, which, in turn, contribute to the plasticity of CSCs [111].

As mentioned above, radioresistance of CSCs is due to their ability to remain dormant [61,75] and to intrinsic recovery mechanisms including DNA repair, the presence of checkpoints in the cell cycle phase where the radiation and routes of survival such as Hedgehog, Notch, and Wnt/β-catenin [61,64] as well as to extrinsic mechanisms from the TME [64] such as tumor hypoxia [108]. However, CSC quiescence is reversible and can therefore result in tumor relapse or metastasis as quiescent CSCs have not lost their division ability and may initiate tumor repopulation, for example, as a direct result of the treatment stress response [61].

As with the tumor itself, TME also responds to RT by inducing the release of pro-inflammatory cytokines and other molecules [126,127], including PDGF, IL1β, TNFα, TGFβ, C-X-C motif chemokine 12 (CXCL12), MMPs, IL6, EGF, and VEGF. Pro-inflammatory cytokines contribute to the upregulation of ROS scavengers in CSC [128] and also to downstream STAT3 signaling activation promoting survival of tumor cells, facilitating tumor regrowth, and leading to the development of highly invasive CSC phenotypes [129,130]. Many of these molecules play an important role not only in mediating the interaction between CSCs and their microenvironment, but are also involved in the regulation of CSC radiosensitivity [129,130,131,132]. These factors generated in an irradiated TME promote survival of tumor and endothelial cells, facilitating tumor regrowth and accelerating the development of highly invasive CSC phenotypes [129,130,131].

Hypoxia is a pathophysiological phenomenon that may occur in the tumor niche when there is a deficient oxygen supply and it is strongly associated with the development and aggressiveness of some solid malignancies and with radioresistance [133]. The hypoxic niche also protects CSC as hypoxia increases radioresistance [134,135,136] favoring early relapse after RT. Hypoxic niches are characterized not only by the lack of oxygen that results in low ROS levels but also by upregulation of ROS scavengers [61,82,128]. Overexpression of ROS scavengers in CSCs, mediated by genes such as superoxide dismutase, superoxide reductase, glutathione peroxidase, and catalase protects themselves from ROS-induced damage, which may contribute to tumor radioresistance [65,101]. In addition, the enhanced mitochondrial respiratory capacity of CSCs prevents ROS overloading, leading to low levels of ROS and protecting CSC from RT-induced cell death [64,81]. CSCs are equipped with efficient oxidant/antioxidant machinery, and treatment strategies to overcome hypoxia-driven radioresistance have always targeted this cancer cell population.

Cell responses to hypoxia are commonly regulated by the hypoxia inducible factor (HIF-1) whose increases inside tumors have been correlated with radioresistance, progression and metastasis [137]. Hypoxia causes activation of HIFs, which enable cells to adapt to the low-oxygen environment. The activation of the hypoxia-inducible factor (HIF) signaling pathway promoted by ROS scavengers [138,139] triggers pro-survival routes associated to radioresistance and accelerated repopulation of CSC during or after RT such as Notch, WNT, and Hedgehog [4,140,141,142,143,144,145].

It can be hypothesized that CSC heterogeneity varies between different niches and depending on the oxygen tension. Thus, in hypoxic conditions, they remain as quiescent radioresistant CSC, and in oxygenated situations, CSCs can proliferate and migrate.

## 5. Radiocurability and Radiation Therapy Resistance

Radiation resistance of CSCs may be either primary (or intrinsic) or acquired mediated by adaptive responses leading more, aggressive, and invasive tumors [146]. It is known that RT preferentially kills non-CSC, thereby increasing the number of CSCs within the tumor [147], which can be used to predict the radiation dose needed to have successful treatment. Consequently, tumors with a higher proportion of CSCs given that the dose of irradiation will have a lower probability of local control in comparison to tumors with fewer CSCs [148,149]. An essential issue in radiobiology is to know the number of CSCs left after RT as survival of one single CSC can lead to RT failure and tumor relapse [150,151]. Tumor radiocurability inversely correlates with tumor volume [152] and with intrinsic radiosensitivity in vitro [153] implying dose-volume dependence. It is important to consider that many patients can be cured with conventional RT, which is in part probably due to the CSC differences between tumor types.

### 5.1. The Five Rs of Radiotherapy

RT remains a feasible approach to treat various cancers. Improvements in medical imaging and technological dose delivery have opened a new horizon in three-dimensional conformal treatment. To better comprehend the impact of dose fractionation on tumor control and organ sparing, radiobiological studies have revealed five principles influencing tissue response to conventionally fractionated radiotherapy, namely: intrinsic radiosensitivity, cell redistribution in the cell-cycle, DNA damage repair, repopulation, and reoxygenation of hypoxic regions (Figure 2).

#### 5.1.1. Radiosensitivity

Radiosensitivity is an intrinsic property of tumor cells [154], which need consideration to obtain the highest curability rates while maintaining damages to normal tissues to the lowest levels [155]. The cells within a tumor exhibit different sensitivity to RT and, consequently, not all subpopulations are equally affected by this treatment. The intrinsic resistance of CSCs associated to their epigenetic plasticity and genetic evolution and might be involved in recurrence and disease progression after irradiation. Radiosensitizers can be used to overcome the intrinsic radioresistance of CSCs and understanding the differing radiosensitivity of tumor cell subpopulations, especially the CSC subpopulation, is vital in order to develop new or improve existing anti-cancer therapies. It has been documented that both, intrinsic radiosensitivity and density of CSC correlate with local tumor control probability after RT [156].

#### 5.1.2. Redistribution Through the Cell Cycle

Tumor cells exhibit differential radiosensitivity depending on the cell cycle phase. In mitosis they are more sensitive to radiation whereas in late S-phase they are more radioresistant [157,158]. Cells that survive the first dose of radiation will progress into a more sensitive phase and therefore, dose fractionation will allow redistribution of radioresistant S-phase tumor cells into a more sensitive phase of the cell cycle. This fact will provide a therapeutic benefit for slowly cycling normal cells [159].

It is well known that in a large proportion of tumor cells, one or more of the cell cycle checkpoints are disabled due to genetic changes and other alterations that occur during tumorigenesis. When functional, the checkpoints block further proliferation of tumor cells and can thus actively suppress cancer development. Alteration in genes that influence checkpoint activation will result in the failure to delay cell-cycle progression in response to irradiation.

The presence or absence of checkpoints will affect the redistribution of cells in the cell cycle after irradiation. Since not only radioinduced damage varies during the course of the cell cycle but also the DNA repair capacity changes, this may indirectly affect the sensitivity of cells to subsequent doses of radiation. A redistribution of the cells into the cell cycle is induced after radiation exposure resulting in an accumulation of the most resistant cells in the S phase. Thus, if the radiation dose is administered at the most sensitive stage, i.e., G2/M, the treatment will be most effective. Quiescence or slow cycling state of CSCs is associated with relative radioresistance [160].

Radiation-induced activation of the DNA damage checkpoint-Chk1 signaling CSCs within non-small cell lung carcinoma (NSCLC) led to cell cycle arrest, more efficient DNA damage repair and a higher cell survival rate. Activation of ATR-Chk1 and ATM-Chk2 signaling pathways and DNA repair are more efficient in CD133+ CSCs, but not in CD133− cells in response to radiation-induced genotoxic stress [5]. Chk1 knockdown in CD133+/CD44+ prostate CSCs abrogated the radiation-induced cell cycle arrest and conferred CSC radiosensitization [161]. An increased radioresistance of the breast CD44+CD24−/low CSCs compared to the non-CSC subpopulation was due to the ATM/Chk2 signaling activation. It must be considered that the efficacy of targeting the ATM and ATR pathways is likely to depend on the genetic context. In this sense treatment with ATM inhibitor KU55933 restored radiosensitivity of the BCSC subpopulation, suggesting that targeting ATM signaling could be effective to eradicate of radioresistant breast cancer cells [162].

The Notch pathway is also involved in the acute response to irradiation triggering the transcription of gene involved in the progression into the S-phase. In breast cancer, irradiation induces the expression of surface Notch receptor ligands in non-tumorigenic cells and the activation of Notch signaling in CSCs that redistribute quiescent CSCs into the cell cycle [58]. Knockdown of the Notch pathway has resulted in radiosensitization of breast cancer cells, leading to cell death especially in CD44+ than in CD44- cells [163]. Fractionated irradiation promotes the recruitment of CSCs from their niche and increases the proportion of cycling cells [164] enhancing their radiosensitivity. In BCSCs, multiple fractions of irradiation alter the kinetics of CSCs since Notch/Jagged expression were far greater than those of single doses [3].

#### 5.1.3. Repopulation of Surviving Normal and Malignant Cells Between Dose Fractions

During an extended course of RT, cells that survive irradiation may proliferate and this repopulation is the main reason for the failure of conventionally fractionated RT [165].

Activation of the Notch, WNT, and Hedgehog signaling pathways is essential not only for CSC maintenance and radioresistance as described above, but also for accelerated repopulation of CSC during or after treatment in glioma, breast cancer, and prostate cancer [4,143,144]. Several studies have shown an increase in CSC phenotypes after repeated, clinically relevant doses of radiation in vitro and in vivo [3,4,5].

#### 5.1.4. Repair of Radioinduced DNA Damage

Cell killing by ionizing radiation is based on the production of unrepairable lesions involving DNA double-strand breaks (DSBs). Most radiation-induced DNA damage is however sublethal, which means that can be repaired, particularly, at lower doses, while at higher doses accumulation of sublethal lesions also contributes to lethality. Critical normal tissues and tumors often differ in their ability to repair radiation damage and thus sublethal damage repair between radiation fractions is exploited in RT. Most of the radio-induced DNA damage is caused by free radicals such as ROS generated by ionization of water molecules, i.e., indirect action of radiation. Thus, free radical scavengers, such as glutathione located near the DNA, play a major role in determining the extent of initial radiation-induced DNA damage and cell survival.

An increased capability for DNA-repair and for scavenging of ROS has been reported for CSCs of breast cancer [166], glioblastoma [5], and lung cancer [167].

The low ROS levels before and after irradiation of murine and human BCSCs was documented by Diehn et al. using primary breast cancer cultures. They also described an antioxidant gene expression profile for BCSCs [168]. This could explain why repeated fractions of radiation preferentially kill the less tumorigenic cancer cells and the enrichment of CSCs [3].

The histone H2AX phosphorylation (γ-H2AX) is an indicator of DNA DSB recognition and repair [169]. Radiation induced few [3] or significantly less [170] γ-H2AX foci in human BCSCs, and in murine BCSCs they resolved faster than in non-CSC populations [4]. In glioma, γ-H2AX foci (DNA DSBs) were repaired more efficiently and more rapidly than their non-CSC counterparts [5]. RAD51 is a protein involved in DSB homologous recombination repair [171] and it is part of the CSC molecular signature in breast cancer [172] suggesting that this repair mechanism may be more important in CSCs.

Repair of slowly proliferating, late-responding tissue after irradiation is favored by the dose fractionation. The outcome of radiation exposure will depend upon the extent of DNA damage and repair but also the signaling pathways involved in the DDR such as cell cycle arrest and cell death will be important. The influence on radiation response of cancer-associated mutations, DNA repair, and cell cycle arrest should be taken into account depending on different individuals. Moreover, since CSCs may differ in the way they handle the DDR it should be considered to determine the outcome of fractionated RT [58].

#### 5.1.5. Reoxygenation

Oxygen levels inside tumors play an important role in the radiation response since the partial oxygen pressure enhances the cell killing effect because radiation-induced ROS. Therefore, the peripheral well-oxygenated areas of tumors are killed first, followed by vascularization of the central tumor along with tumor shrinkage. Repetition of this process enhances the efficacy of RT.

After a first dose of radiation surviving cells will tend to be hypoxic but thereafter their oxygen supply may improve, leading to an increase in radiosensitivity. Human tumors contain zones of acute and chronic hypoxia often associated with poor prognosis because of local recurrence or systemic disease [173]. Intermittent vessel occlusion induces transient areas of acute hypoxia that may reoxygenate rapidly, whereas limitation of oxygen diffusion generates areas of chronic hypoxia secondary that difficult reoxygenation. By decreasing RAD51-mediated homologous recombination DNA repair chronic hypoxia may make cells radiosensitive [172]. Considering this fact, cells undergoing intermittent hypoxia are candidates for therapy resistance. Thus, having a deeper understanding of the response of CSCs to irradiation under varying hypoxic conditions may help to determine the effects of both hypoxia and irradiation in selecting for CSCs [3,164].

The 5Rs are useful for the identification of tumor and environmental conditions determining radioresistance or radiosensitivity in tumors and take into account the process of tumor contraction. Of these five parameters, intrinsic radiosensitivity may be measured before treatment in contrast to the rest of the parameters that may be obtained during treatment. Repair and repopulation will confer resistance to the tissue to a second dose of radiation; redistribution and reoxygenation are likely to make the tissue more sensitive to a subsequent dose. Therefore, repopulation of tumors and regeneration of normal tissue are critical in the success of fractionated RT. The relationship between the 4Rs of RT and radioresistance acquisition has been summarized recently by Sato et al. [174].

### 5.2. CSCs: Targets for Radiosensitization

Radioresistance of CSC involves the administration of higher irradiation doses than the usual doses for their eradication, as higher doses increase the probability of CSC eradication. Using a xenograft model, our previous research work has shown that higher irradiation doses (6 Gy) are associated with tumor growth delay, probably due to CSC apoptotic death [96]. Saga [175] has suggested that in order to avoid recurrence or metastasis in prostate cancer the administration of higher doses would be necessary to eliminate the radioresistant CSCs. In addition to the cytotoxic effects, radiation plays a role in immune modulation in the tumor and TME. Radiation increases the antigen-specific antitumor immune responses through various proposed mechanisms. Clinical trials investigating immune checkpoint blockade (ICB) and SBRT are being explored [176]. High-dose ablative RT may be more effective in causing an abscopal effect than fractionated RT [177].

In addition to photons, protons can be used to eradicate CSCs [178,179,180,181,182,183,184]. Photons and protons with low LET are considered to have a similar relative biological effectiveness (RBE) but some authors have reported different biological effects between proton and photon irradiation [185]. Recent research shows that there are some advantages in using particle therapy compared to conventional photon RT, the most important one is the optimal dose distribution [180] with energy deposition specifically focused on the tumor in order to spare normal tissue and allowing the delivery of higher radiation doses to the target tissue.

Compared to photons, proton beam irradiation increased ROS levels, induced more single and double strand DNA breaks, less DNA damage repair (as measured by H2AX phosphorylation), and led to increased apoptosis by decreasing cell cycle recovery [186].

In addition, proton irradiation increases the sensitivity of CSC from different cell lines, such as breast or prostate cancer, to cytotoxic T-cell killing [187]. These findings encourage one to further explore a combined use of proton irradiation with immunotherapy.

On the other hand, new high LET radiation therapy modalities have emerged as contributors to overcome CSC-related resistance, such as boron-neutron capture therapy (BNCT) and carbon-ion particle therapy [188,189], used alone or in combination with other targeted treatments like PARP inhibitors [190,191,192,193]. Since BNCT releases high-LET radiation, it should provide RBE and a lower oxygen enhancement ratio (OER), compared to conventional RT. Thus, BNCT could be an effective strategy for the treatment of radioresistant tumors, such as clear cell sarcoma (CSS) [194] and osteosarcoma [195]. Clinical trials using BNCT on patients with head and neck, brain, lung, and liver cancers have shown promising results in terms of overall survival, recurrence, and metastasis [196]. Primary human glioma stem cells that were resistant to photon treatment could benefit from carbon ion irradiation as this therapy impairs the capacity to repair carbon ion-induced DNA double strand breaks [186]. Several clinical trials with carbon-ion radiotherapy for malignant tumors have also shown promising results [197,198,199,200,201,202].

In comparison to photon irradiation that is strongly affected by oxygen levels for the induction and maintenance of DNA damage, however particle irradiation has low dependence on tissue oxygenation. Thus, high LET particle beams are less affected by the hypoxic conditions often found in solid tumors [203]. Additionally, high LET radiation induces similar RBE than a photon with lower doses. Lastly, the tumor control rates of high LET RT may be improved with the use of CSC-targeted therapy and further studies are warranted to establish optimal combinations.

### 5.3. Therapeutic Targeting of CSCs Metabolism

Altered metabolism is a hallmark of cancer and the target of novel therapeutic strategies. As with normal tissues, tumors contain metabolically different cell populations. Some authors have proposed the term “energetic” CSCs in breast cancer with a hyperproliferative phenotype [204]. Taking into account the redox state, metabolic heterogeneity has been associated to differences in stemness properties and to radioresistance [168].

Metabolic reprogramming has shown to delay CSC differentiation. Unlike normal cells that use oxidative phosphorylation (OxPhos), cancer cells use glycolysis, whereas CSCs show a particular metabolic flexibility driven by environmental stimuli, switching between glycolysis and OxPhos to maintain homeostasis and promote tumor growth [66]. For instance, glioma stem cells rely on OxPhos, but switch to glycolysis when oxidative metabolism is inhibited in the presence of oxygen [205]. Glycolysis facilitates survival and fast adaptation to hypoxic tumor environment avoiding toxic ROS accumulation. Another advantage of glycolysis in tumor cells is the generation of an acidic environment that promotes invasion and suppresses the immune response [206,207]. Moreover, glycolytic metabolism sustains stemness in normal stem cells and CSCs of several cancer types [208]. Different studies have supported the glycolysis dependence of CSCs in several types of cancer [209,210,211] as in radioresistant nasopharyngeal carcinoma, whose stage-specific embryonic antigen (SSEA)-expressing cells usually appear as compacted spheres [212]. On the other hand, OxPhos has been described as the main source of energy in CSCs from a number of cancer types [213,214,215,216,217]. Mitochondrial metabolism coupled to OxPhos constitutes a more efficient energy process in CSCs, which it would make a better use of limited nutrients [215]. It is also important to consider that stromal cells release a variety of metabolites that can be used by OxPhos-dependent cells conferring them with increased adaptability to the changing conditions of the tumor microenvironment [218].

Cell plasticity through EMT has also been linked to metabolic reprogramming as a relevant step of CSCs during metastasis. Thus, some authors have shown that distinctive metabolic programs have been displayed for organ-selective metastatic breast cancer cells [219]. The specific energy requirements of CSCs during metastasis may represent a therapeutic window in the late stages of disease.

Inhibition of metabolic pathways is a new strategy to fight glioblastoma. It has been shown in freshly isolated glioma stem cells that dichloroacetate (DCA), an orphan drug, has the capacity to switch the metabolism from mitochondrial OxPhos to cytoplasmic glycolysis, which in turn increases mitochondrial ROS and induces apoptosis. Moreover, glioblastoma patients treated with RT and temozolomide receiving DCA showed a slower tumor progression [220]. Thus, the combination of treatments that inhibit the main metabolic pathways would eradicate the CSC subpopulations within the tumor consequently reducing the risk of resistance and relapse [221]. Further research is needed to better understand the metabolic reprogramming process.

### 5.4. Targeting Redox Homeostasis

ROS and redox signaling play an important for CSCs functionality. Low ROS niches support stemness characteristics of quiescent stem cells, in contrast the increase of ROS content promotes stem cell proliferation and differentiation [222,223]. ROS levels are regulated by CSCs via antioxidant transcription factors, such as NRF2 or FOXO [168,224,225], which affect redox homeostasis through direct or indirect modulation of cellular metabolism.

Most of the radioinduced damage is an indirect action via the production of free radicals, mainly ROS. Thus, the main cytotoxic effect of RT results from the accumulation of intracellular ROS that induces cancer cell death. This approach may not be effective against CSCs because of their increased antioxidant potential [168,226]. On the other hand, ROS promotes CSC survival and invasion by acting as signaling molecules [227].

Glutathione is an important ROS scavenger that plays a crucial role in the maintenance of stemness characteristics [168]. In this sense, the blockade of glutathione synthesis could be a novel therapeutic approach for the eradication of the CSC population and reduction of tumor growth. Some authors have shown the efficacy of buthionine sulfoximine (BSO), an inhibitor of glutathione biosynthesis, in decreasing clonogenicity and enhancing the response of CSCs to RT [168,228,229]. Lastly, the inhibition of enzymatic cellular antioxidants has been associated to increased sensitivity of human breast CSCs to RT [229].

## 6. Future Perspectives in Radiation Oncology Targeting CSCs: Novel Treatment Approaches

Targeting strategies to increase CSC radiosensitivity open a therapeutic window between tumor control and the induction of adverse reactions in normal tissue. The combination of both approaches should allow the development of more opportunities for a successful multimodality treatment of cancer.

The concept of dynamic theory of CSCs explains tumor recurrence after initially successful RT, the phenomenon of tumor dormancy, and metastasis. The concept of CSC has fueled the development of innovative therapeutic strategies aimed not at shrinking tumor bulk, but rather at eradication of CSCs, which are responsible for long-term growth. The inactivation of even only a limited number of CSC might significantly improve local tumor control [150]. Nevertheless, CSC plasticity makes difficult the identification and eradication of CSCs since a non-CSC may gain CSC traits and repopulate the tumor after treatment. Modulation of stem cell–niche functions is likely more attractive than to pursue therapies that are based on intrinsic CSC features. Therefore, a deep insight into the foundations of cell plasticity in normal and tumor cells is essential for the development of smarter CSC targeting therapies [81,230,231].

The mechanism of tumor regrowth involves the continuing proliferation of CSCs, as it has been shown in brain, skin, or intestinal tumors [232]. Functional imaging, as a non-invasive, quantitative method permits the evaluation of the regional heterogeneity in a tumor during RT. ^18^F-FLT PET is a useful tool in oncology for estimating changes in tumor proliferation and thus repopulation during RT, with high specificity and sensitivity [233].

The identification of specific biomarkers of CSC subpopulation in pre-treatment biopsies and during the course of tumor treatment is a challenging strategy for generation of refined targeted therapies. The identification of CSC-related biomarkers might help in tumor diagnosis and staging, treatment selection as well as in the determination of patient prognosis and prediction of patient response to RT.

Some inherent technical and conceptual limitations have been associated to the xenotransplantation approach to investigate the properties of CSCs (i.e., the lack of a functional immune system in the host mice is a limitation for clinical translation). Several research groups have studied CSCs in intact tumors through genetic-lineage tracing for assessing adult stem cell activity in situ for more direct evidence [26]. Experiments using lineage tracing and cell ablation in intact tumors confirmed the presence of stem cells in dedicated niches [234,235,236,237]. In addition, promising approaches for eradication of CSCs centered in the modification of the TME, by reoxygenation of CSC niche, reactivation of cytotoxic T cells or targeting tumor-associated macrophages and fibroblasts have been explored [238].

CSCs display many features of embryonic or normal tissue stem cells, activating highly conserved signal transduction pathways involved in organs development and tissue homeostasis, including the Notch, Hedgehog (Hh), and Wnt pathways. Moreover, CSCs generally have slow growth rates and are more resistant to chemotherapy and RT than differentiated tumor cells [239,240]. Thus, new therapeutic options targeted against these pathways have been developed.

Indeed, CSCs residing in fibrotic tissue and other microenvironmental niches can escape from the effects of conventional cytotoxic treatments [241] and they can resume after treatment cessation, driving to recurrence in patients. Thus, treatments targeting the CSC population, and therefore their primary or acquired radioresistance, could dramatically transform life expectance and treatment outcomes in oncology. In fact, several new drugs targeting the Notch, Hedgehog, and Wnt pathways have entered clinical trials [242,243].

Both, the Notch and Hedgehog signaling can involve canonical and non-canonical pathways. Targeting Notch signaling affects several cell types within tumors such as CSCs, immune cells, vascular endothelial cells, and differentiated tumor cells. Several types of Notch-pathway inhibitors are in clinical development. Particularly, GSIs in combination with whole brain RT or stereotactic radiosurgery has been investigated in patients suffering from brain metastases (NCT01217411). GSIs have also been used in combination with RT and temozolomide in glioma patients (NTC01119599). Inhibition of histone deacetylases (HDAC) could suppress tumor growth and induce apoptosis of tumor cells via upregulation of Notch1 [244]. Another trial has studied the effects of the same HDAC inhibitor in metastatic medullary or radioiodine-resistant differentiated thyroid cancers (NCT01013597) [245]. These approaches will require the consideration of all cross-signaling networks in CSCs in a context-dependent manner and specific disease settings. In fact, Notch signaling can interface with and influence-relevant pathways involved in the cell cycle, apoptosis, EMT, and DNA repair [246].

The Hedgehog signaling pathway is involved in tissue patterning during embryonic development and the repair of normal tissues, and EMT process [247]. There are either autocrine or paracrine mechanisms targeting this pathway. The most clinically advanced agent targeting the Hedgehog pathway is vismodegib. Two clinical assays have been conducted using this drug in combination with hypofractionated (NCT02956889) and conventional RT (NCT01835626) in patients with advanced basal cell carcinoma of the head and neck but no results have been posted on the ClinicalTrials.gov website as of yet.

The Wnt signaling cascade comprises of three major pathways: the tumorigenic canonical Wnt pathway, which involves activation of β-catenin–T-cell-specific/lymphoid enhancer-binding factor (TCF/LEF) transactivation complex; the non-canonical planar-cell polarity pathway that regulates the cytoskeleton; and the non-canonical Wnt–calcium pathway, which regulates intracellular calcium levels [248]. Anticancer therapies targeting the canonical Wnt signaling are in development but, to our knowledge, there are no clinical trials combining these drugs with RT.

Considering EGFR inhibitors, the greatest clinical experience so far has been reported for head and neck cancer when EGFR is highly expressed. It has been shown that EGFR inhibition might counteract mechanisms of intrinsic radioresistance. Retrospective analyses from the DAHANCA (Danish Head and Neck Cancer) and the CHART (continuous hyperfractionated accelerated radiotherapy) trials have shown that only patients with high EGFR expression benefited from accelerated radiotherapy [249,250]. These data suggest that the EGFR may be involved in accelerated repopulation of CSCs. Another factor involved in radioresistance is the enrichment in CSCs after repeated X-ray irradiation. Consequently, low LET radiation treatment can increase the number of CSCs [251,252] by promoting asymmetric cell proliferation. Some authors have suggested that the accumulation of DNA damage by repeated X-ray irradiation favors both EMT and CSCs enrichment, which increase oncogenic activity. Secondary induction of a CSC subpopulation by EMT also contributes to the induction of radioresistance [174]. The differences between photon and particle irradiation concerning the effects of repeated in vivo irradiation remain largely unknown. Repeated high doses of X-ray in vitro can induce significant resistance to both X-ray and carbon-ion radiation. Although evidence is limited to a single tumor cell type, some results have shown that contrary to repeated in vitro irradiation, regrown in tumor models after repeated photon or carbon-ion irradiation does not increase their radioresistance. However, repeated photon irradiation but not particle irradiation enhances metastasis and increased aggressiveness of regrown tumors [253]. Very few clinical trials have been conducted involving CSC and RT (Table 1). Moreover, since microRNAs are epigenetic gene regulators associated with tumor initiation and progression, they have been recently considered as promising therapeutic targets modifying the therapeutic resistance of CSCs through epigenetic modification [95,254].

Although tumor dedifferentiation is strongly regulated by genetic and epigenetic factors, exogenous signals from the TME also have an important role. In several solid tumors, several studies showed that differentiated non-cancer stem cells are able to reacquire stem cell characteristics under irradiation stress [255,256]. Moreover, dying cells and their released damage associated molecular patterns (DAMPs) are major components of TME after RT. Some authors have proposed a strategy that combines inhibitors of a DAMP molecule, HMGB1, with radiation for synergistic treatment of pancreatic carcinoma [257].

RT can eradicate cancer cells while simultaneously triggering the release of pro-inflammatory mediators and increasing tumor-infiltrating immune cells [126,127]. Thus, concerning the microenvironment targeting, RT has been proposed as an adequate treatment with immune checkpoint inhibitors (ICIs) and other immune-oncology treatments [258,259,260,261,262,263]. RT promotes the conversion of immunologically ‘cold’ into ‘hot’ tumors. Moreover, the immunomodulatory effect of RT has been associated to tumor cell-intrinsic events driven by DNA damage [264,265].

## 7. Conclusions

Taken together, current findings suggest that CSCs contribute to tumor radioresistance and a better understanding of the molecular mechanisms underlying CSC plasticity and interaction with their niche could improve cancer treatment. Thus, combination therapy with a charged particle and molecular CSC-targeting is crucial to overcome radioresistance [266]. Approaches targeting TME also need to be developed. Additionally, considering the inflammatory TME induced by RT, enhancing the immunogenic effects of RT through DDR inhibitors while inhibiting immunosuppressive aspects through ICIs would be a promising therapeutic strategy. RT induces immunogenic cell death also contributing to the achievement of systemic tumor control through the abscopal effect. More research is needed regarding the great potential of particle therapy in radioresistant cancers. Lastly, a special interest is needed in the evaluation of the impact of carbon ion and proton irradiation on eradication of CSCs, their role in hypoxia, and the identification of targetable pathways in combination with particle radiation.

## Figures and Tables

**Table 1:** Clinical trials combining CSC and RT.

Identifier	Tumor Type	No. of Patients	Phase/Status	Treatment Schedule	Toxicity/Adverse events (Serious/Not Serious)	Results
**NCT01868503**	Locally advanced or locally recurrent breast cancer that is refractory to chemotherapy	7	II/Terminated (protocol modification)	Conventional RT + Lapatinib ditosylate	Lymphocytes count decreased/anemia/ fever-possible sepsis/endocrine disorders	Change in the proportion of BCSCs not analyzed
**NCT02039778**	Brain tumor	4	Not applicable/ Terminated (poor accrual)	Stem cell RT/IMRT + Temozolamide	Death^†^/platelet count decreased^†^/blurred vision^†^/fatigue^†^/ nausea^†^/ headache^†^/dry skin^†^	Not completed
**NCT04031378**	Oligometastatic prostate	100	II/Not yet recruiting	Single Dose RT (SDRT) with or without adjuvant systemic therapy	Not provided	No results posted
**NCT03085992**	Resectable rectal cancer	49	II/Completed	FOLFOXIRI ^†^ Bevacizumab Chemoradiotherapy ^†^ Bevacizumab	Not provided	No results posted

**^†^** Indicated events were collected by systemic assessment.

## Abbreviations

ABC	ATP-binding cassette	^18^F-FLT	^18^F-fluorothymidine	NSCLC	non-small cell lung carcinoma
Akt	protein kinase B	FGF	fibroblast growth factor	Oct-4	octamer binding protein 4
ALDH	enzyme aldehyde dehydrogenase	FOXO	forkhead Box class O	OER	oxygen enhancement ratio
APLN	angiopoietins, ephrins, apelin	γ-H2AX	phosphorylated Histone 2AX	PDGF	platelet-derived growth factor
ATM	ataxia-telangiectasia mutated	GS	γ secretase	PET	positron emission tomography
ATR	ataxia telangiectasia and Rad3-related protein	GSIs	γ-secretase inhibitors	PlGF	placental growth factor
Bcl-2	B-cell lymphoma 2	GSK-3β	glycogen synthase kinase 3 beta	PTEN	phosphatase and tensin homolog
BCSC	breast cancer stem cells	HDAC	histone deacetylases	PI3K	phosphatidylinositol 3-kinase
BSO	buthionine ulfoximine	HGF	hepatocyte growth factor	RAD51	
CAFs	cancer-associated fibroblasts	Hh	hedgehog signaling	ROS	reactive oxygen species
CAIX	carbonic anhydrase IX	HIF1a	hypoxia-inducible factor-1a	RBE	relative biological effectiveness
CDC20	cell-division cycle protein 20	HR	homologous repair	ROS	reactive oxygen species
CD133	prominin I	ICB	immune checkpoint blockade	RT	radiotherapy
CHART	continuous hyperfractionated accelerated radiotherapy trial	ICIs	immune checkpoint inhibitors	SBRT	stereotactic body radiation therapy
chks	checkpoints	IL1β	interleukine 1β	SSEA	stage-specific embryonic antigen
CHK1	checkpoint kinase 1	IL4	interleukine 4	SP	side population
CHK2	checkpoint kinase 2	IL6	interleukine 6	TAMs	tumor-associated macrophages
CSC	cancer stem cells	IL8	interleukine 8	TANs	tumor-associated neutrophils
CTC	circulating tumor cells	IL10	interleukine 10	TIMPs	endogenous metalloproteinase inhibitors
CXC	C-X-C motif chemokines	LET	linear energy transfer	TGF-ß	transforming growth factor-ß
CXCL12 C-X-C	motif chemokine ligand 12	M1	classically activated macrophage	TGF-ß1	transforming growth factor-ß1
DAHANCA	Danish Head and Neck Cancer trial	M2	alternatively activated macrophage	TNF-α	tumor necrosis factor-alpha
DAMPs	damage associated molecular patterns	MDSC	myeloid-derived suppressor cells	TICs	tumor initiating cells
DCA	dichloroacetate	MiRNAs	microRNAs	TME	tumor microenvironment
DDR	DNA damage response	MMPs	matrix metalloproteases	TRAIL	Fas receptor and TNF-related apoptosis inducing ligand
DNA	deoxyribonucleic Acid	mRNA	messenger RNA	Treg	regulatory T cells
DNA-PKc	DNA-dependent protein kinase, catalytic subunit	mTOR	mammalian target of rapamycin	TSCs	tumor stem cells
DSBs	double-strand breaks	NFκB	nuclear factor-κB	UPR	unfolded protein response
ECM	extracellular matrix	NHEJ	non-homologous end joining	UV	ultraviolet
EGF	epidermal growth factor	NRF2	nuclear factor-erythroid 2 p45-related factor 2	VEGF	vascular endothelial growth factor
EMT	epithelial-mesenchymal transition	NOD/SCID	nonobese diabetic/severe combined immunodeficient	Wnt	wingless/integrated
